# The Association between Psychological Distress and Self-Reported Sleep Duration in a Population-Based Sample of Women and Men

**DOI:** 10.1155/2015/172064

**Published:** 2015-11-29

**Authors:** Timothy J. Cunningham, Anne G. Wheaton, Wayne H. Giles

**Affiliations:** Division of Population Health, National Center for Chronic Disease Prevention and Health Promotion, Centers for Disease Control and Prevention, 4770 Buford Hwy, Mailstop F78, Atlanta, GA 30341, USA

## Abstract

Mental health and sleep are intricately linked. This study characterized associations of psychological distress with short (≤6 hours) and long (≥9 hours) sleep duration among adults aged ≥18 years. 2013 Behavioral Risk Factor Surveillance System data (*n* = 36,859) from Colorado, Minnesota, Nevada, Tennessee, and Washington included the Kessler 6 (K6) scale, which has been psychometrically validated for measuring severe psychological distress (SPD); three specifications were evaluated. Overall, 4.0% of adults reported SPD, 33.9% reported short sleep, and 7.8% reported long sleep. After adjustment, adults with SPD had 1.58 (95% CI: 1.45, 1.72) and 1.39 (95% CI: 1.08, 1.79) times higher probability of reporting short and long sleep duration, respectively. Using an ordinal measure showed a dose-response association with prevalence ratios of 1.00, 1.16, 1.38, 1.67, and 2.11 for short sleep duration. Each additional point added to the K6 scale was associated with 1.08 (95% CI: 1.07, 1.10) and 1.02 (95% CI: 1.00, 1.03) times higher probability of reporting short and long sleep duration, respectively. Some results were statistically different by gender. Any psychological distress, not only SPD, was associated with a higher probability of short sleep duration but not long sleep duration. These findings highlight the need for interventions.

## 1. Introduction 

Two Healthy People 2020 goals are to increase public knowledge of how adequate sleep improves health and to improve mental health through prevention [1]. According to* Healthy People 2020*, “mental health is a state of successful performance of mental function, resulting in productive activities, fulfilling relationships with other people, and the ability to adapt to change and to cope with challenges” [1]. Previous studies suggest that the prevalence of SPD (a nonspecific measure that incorporates symptoms of anxiety, depression, and other types of psychological distress) among US adults is between 3.2 and 5.4% [2–6]. Both inadequate sleep and poor mental health are major causes of employee absenteeism and impaired work productivity [7, 8]. Since 1985, the mean age-adjusted sleep duration among US adults has declined. In 1985, 65.9% of adults reported sleeping 7 to 8 hours in a 24-hour period, compared with 62.3% in 2012 [9]. Therefore, mental health and sleep remain major public health priorities.

The association between mental health and sleep has been the focus of health research for over 100 years [10]. Sleep problems are symptoms of depression and anxiety and associated with psychosis according to the fifth edition of* the Diagnostic and Statistical Manual of Mental Disorders* (DSM-5) [11]. Furthermore, studies suggest that impaired sleep may directly contribute to the development of some psychiatric disorders [12–15]. Insomnia, a disorder characterized by difficulty falling or staying asleep for example, has been shown to increase the likelihood of subsequent depression [16, 17]. On the other hand, some studies have shown bidirectional associations and other studies have shown that poor mental health may raise the risk for impaired sleep [18, 19]. In particular, two previous studies have found associations between sleep duration and serious psychological distress, as measured by the Kessler 6 (K6), which is a 6-question measure of nonspecific psychological distress developed for large, representative, health surveys and predictive of serious mental illness [13, 14]. To the best of our knowledge, no study has investigated the possible presence of dose-response associations and the functional form of the associations of psychological distress with short and long sleep duration in addition to associations of SPD among adults using the K6. Furthermore, it remains unknown whether these associations are modified by gender.

To address these gaps, we analyzed population-based data from women and men in the 2013 Behavioral Risk Factor Surveillance System (BRFSS). First, the associations of SPD with short and long sleep duration are examined. Second, the associations of a five-category ordinal measure of psychological distress with short and long duration are examined. Third, associations of a scale measure of psychological distress with range 0 to 24 with short and long duration are examined. Our* a priori* hypotheses were that SPD would be associated with a higher probability of both short and long sleep durations. We also hypothesized that there are dose-response associations for short and long sleep duration and differences by gender.

## 2. Methods

### 2.1. Study Population

BRFSS is an annual state-based, random-digit-dialed telephone survey of noninstitutionalized, US adults aged ≥18 years in the 50 states and the District of Columbia (DC) [20]. BRFSS includes questions on sociodemographic characteristics, chronic diseases, health behaviors, and access to health care. In 2013, a new question on sleep duration was included in the core questions and five states (Colorado, Minnesota, Nevada, Tennessee, and Washington) implemented the optional BRFSS mental illness and stigma module and comprised the study sample. The cooperation rate, which is defined as the number of completed interviews divided by the number of eligible respondents who were successfully reached by an interviewer, for each of the five states was 75.9% (Colorado), 73.2% (Minnesota), 72.6% (Nevada), 72.5% (Tennessee), and 51.8% (Washington) [21]. The response rate, which is defined as the number of respondents who completed the survey as a proportion of all eligible and likely eligible persons, for each of the five states was 58.0% (Colorado), 54.3% (Minnesota), 43.7% (Nevada), 45.9% (Tennessee), and 31.1% (Washington) [21]. We analyzed available data from 36,859 US adults aged 18 years or older in five states for the variables included in this study. This study was exempt from human subjects review as the data were obtained from public-use surveillance datasets.

### 2.2. Independent Variables

The K6 scale is included in the optional BRFSS mental illness and stigma module and is based on the Kessler 10 scale of nonspecific psychological distress [22]. Respondents were asked “During the past 30 days, about how often did you feel… (1) nervous, (2) hopeless, (3) restless or fidgety, (4) so depressed that nothing could cheer you up, (5) everything was an effort, and (6) worthless?” Symptom frequencies are described on a 5-point Likert scale with the responses (1) all, (2) most, (3) some, (4) a little, and (5) none of the time [22, 23]. Responses are scored 4 to 0, respectively, and response scores are summed, for a total possible score range of 0 to 24. K6 scores of 13 or greater versus 12 or fewer were used as a cut-point to distinguish people with and without SPD. Additionally, the K6 scores were grouped into five categories (0, 1 to 2, 3 to 5, 6 to 10, and 11 or greater) as defined in previous studies [6, 24]. There was approximately 30%, 30%, 20%, 15%, and 5% of the population in each category.

### 2.3. Dependent Variables

Respondents were asked “On average, how many hours of sleep do you get in a 24-hour period?” Because the National Institutes of Health recommends 7-8 hours of sleep per day for healthy adults (http://www.nhlbi.nih.gov/health/health-topics/topics/sdd/howmuch), self-reported short sleep duration was defined as ≤ 6 hours of sleep in an average 24-hour period and self-reported long sleep duration was defined as ≥ 9 hours of sleep in an average 24-hour period.

### 2.4. Sociodemographic Characteristics

Potentially confounding sociodemographic characteristics considered for these analyses included age group (18–24, 25–34, 35–44, 45–64, or ≥ 65 years), gender (men or women), race/ethnicity (non-Hispanic white; non-Hispanic black; non-Hispanic American Indian or Alaskan Native; non-Hispanic Asian; non-Hispanic Native Hawaiian or other Pacific Islanders; non-Hispanic other races; non-Hispanic multiracial; or Hispanic), marital status (married; previously married including those divorced, widowed, or separated; or never married or members of an unmarried couple), educational attainment (did not graduate high school, graduated high school or obtained the general equivalent degree, attended some college or technical school, or graduated college or technical school), annual household income (<$25,000, $25,000–$49,999, ≥$50,000, or missing), employment (employed, unemployed, homemaker/student, retired, or unable to work), and health insurance coverage (yes or no).

### 2.5. Statistical Analysis

All analyses were conducted using SAS-callable SUDAAN version 11.0 (Research Triangle Institute, Research Triangle Park, North Carolina) to account for complex sampling design of BRFSS. All estimates were weighted to represent the state population. Results were considered significant at *P* < .05. We examined differences in SPD, short sleep duration, and long sleep duration by selected sociodemographic characteristics using Chi-squared tests. Additionally, we examined differences in sleep duration by SPD and a five-category K6 measure using Chi-squared tests. Poisson regression models with robust variance were used to estimate prevalence ratios (PRs) for the probability of reporting short sleep duration or long sleep duration and the corresponding 95% confidence intervals (CIs) for all respondents with complete data and separately for women and men for three specifications of the K6: a dichotomous measure of SPD (cut-point 13), a five-category measure, and a scale measure with range 0–24 [25, 26]. We also examined quadratic terms for the scale measure of psychological distress. The multivariable adjusted regression models included the following relevant covariates: age group, race/ethnicity, marital status, educational attainment, annual household income, employment, and health insurance coverage.

## 3. Results

Overall, 76.3% were non-Hispanic white, 51.5% were women, and 36.7% were 45–64 years (Table 1). The prevalence of SPD was 4.0% (95% CI = 3.7, 4.4) overall and varied by gender: women were more likely to report SPD (*P* < .05). The prevalence of short sleep duration was 33.9% (95% CI = 33.1, 34.7) overall and varied by gender: women were less likely to report short sleep (*P* < .05). The prevalence of long sleep duration was 7.8% (95% CI = 7.3, 8.2) overall and varied by gender also: women were more likely to report long sleep (*P* < .05).

**Figure 1:**
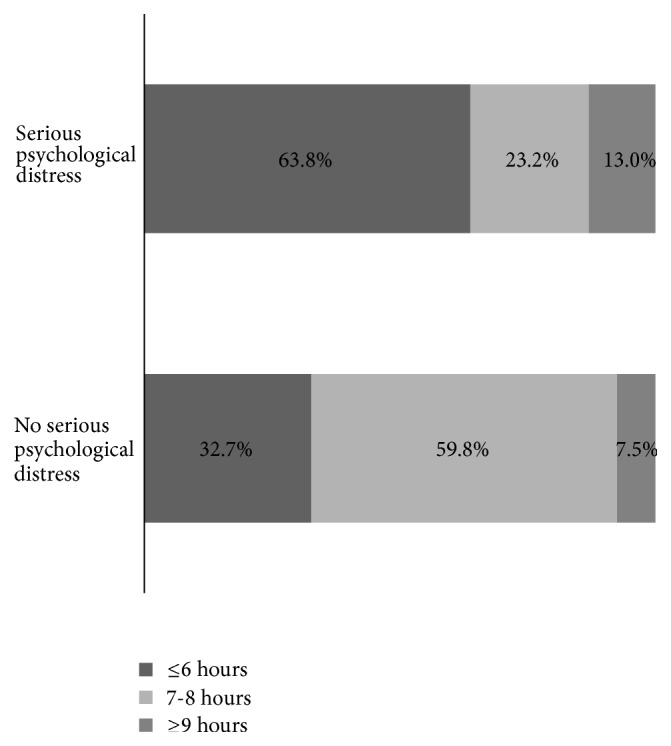
Distribution of average hours sleep in 24-hour period by serious psychological distress among adults aged ≥ 18 years in 5 states, Behavioral Risk Factor Surveillance System, 2013.

The proportion of persons reporting short sleep duration and long sleep duration was higher among people with SPD than those without it (Figure 1; *P* < .05).

**Figure 2:**
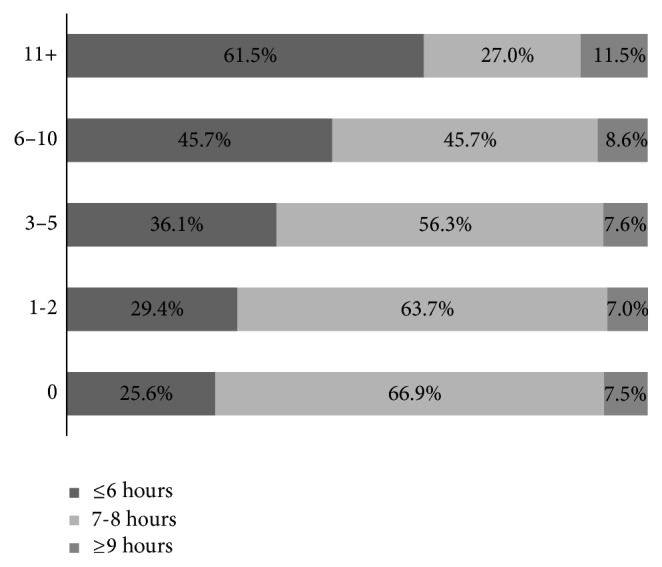
Distribution of average hours sleep in 24-hour period by K6 category among adults aged ≥ 18 years in 5 states, Behavioral Risk Factor Surveillance System, 2013.

The proportion of persons reporting 7-8 hours of sleep on average was lower with increasing K6 categories (Figure 2; *P* < .05) from 66.9% among those with a K6 score of 0 to only 27.0% among those with score of 11+.

In the crude models, SPD, the five-category measure, and the scale measure of psychological distress were associated with a higher probability of reporting short sleep duration (Table 2; *P* < .05). The quadratic term for the scale measure was associated with a lower probability of reporting short sleep duration (Table 2; *P* < .05). These associations for short sleep duration remained statistically significant after adjustment for gender, age, race/ethnicity, marital status, educational attainment, annual household income, employment, and health insurance coverage. We found statistical interactions (*P* < .05) for gender with the five-category measure and the scale measure of psychological distress in association with short sleep duration. This distinction is statistically, but not always practically, significant.

In the crude models, SPD, the five-category measure, and the scale measure of psychological distress were associated with a higher probability of long sleep duration for all respondents (Table 3; *P* < .05). The quadratic term for the scale measure was not statistically significant for long sleep duration. Therefore, it was not included in the final models. After covariate adjustment, the associations for SPD and the scale measure of psychological distress remained statistically significant but not the five-category measure. We did not observe any statistically significant interactions between gender and the three specifications of the K6 in association with long sleep duration.

## 4. Discussion

This study using population-based data demonstrated a strong link between psychological distress and self-reported sleep duration. Furthermore, our study revealed that associations of psychological distress as measured by the K6 scale with short and long duration are not limited to a dichotomous measure of SPD and may vary by gender. The hypotheses were partially supported. We found positive linear associations and nonlinear associations for short sleep duration but only positive linear associations for long sleep duration; the higher the score on the K6 scale, the higher the probability of short and long sleep duration. Additionally, levels of psychological distress below the traditional cut-point for SPD were associated with a higher probability of short sleep duration. That is, we found dose-response associations for short sleep duration, but we did not find dose-response associations for long sleep duration.

### 4.1. Contributions to Extant Literature

These findings are broadly consistent with those of previous studies. In a study of 20,822 Australian young adults (aged 17–24 years) in the DRIVE study, self-reported short sleep duration was associated with prevalent psychological distress and was an independent risk factor for its persistence 1 year later [13]. In another study, among 187,091 US adults aged ≥ 18 years from seven waves of the National Health Interview Survey, participants with high psychological distress scores reported 7-8 hours of sleep less often than those in low or moderate psychological distress and were also most likely to sleep for ≤6 hours or ≥9 hours [14]. Additionally, in a study of adults aged 19–67 years in the first wave and aged 29–80 years in the second wave of the Norwegian Nord-Trøndelag Health Study, among participants not depressed, chronic insomnia increased the risk of developing depression by six times. A similar effect was found for chronic depression (without insomnia) on onset of insomnia [18].

To our knowledge, there are no previous studies that have examined the associations of various formulations of psychological distress with short and long sleep duration by gender. Interestingly, particularly at lower levels, associations with short sleep duration for the five-category measure of psychological distress were more robust among women than men in our study. Additionally, there were gender differences in the associations with short sleep duration for the scale measure of psychological distress that were statistically significant but not necessarily clinically meaningful at the individual level. Nonetheless, the observed gender differences as well as the finding that the prevalence of SPD was less among men than women are consistent with the broader epidemiologic literature [2].

There are likely at least four alternative explanations for these findings [2]. First, psychological distress might be related to gender differences in personality traits or biological components as observed with anxiety and depression [27, 28]. Second, because the K6 does not differentiate well between anxiety and depression, there may be gender differences in the types of psychological distress. Third, women may be more vulnerable or more exposed to risk factors associated with psychological distress [29, 30]. Finally, there are gender differences in the expression of emotions [31, 32]. To the extent that women and men express their psychological distress or emotions in a different way, population-based interventions are likely to be less than optimally effective if this heterogeneity is overlooked. In addition to the higher prevalence of SPD we observed, there is growing evidence that women may be more sensitive to emotions. Further research is needed to examine if these possible explanations apply to gender differences in the association of psychological distress with sleep duration. A greater understanding of gender differences in the associations between psychological distress, emotions, and sleep duration may yield more effective interventions.

The purposes and mechanisms of sleep are complex and not fully elucidated; however, advances in the neurobiology of sleep and mental illness indicate that adequate sleep is critical to optimal mental functioning, productive activities, fulfilling relationships with others, and the ability to adapt to change and to cope with challenges for both women and men [12]. Moreover, inadequate sleep impacts alertness, hormone regulation, memory formation, emotional regulation, executive function, and other aspects of behavior [12].

In addition to SPD, milder psychological distress has strong dose-response and positive linear associations with both emergency room department utilization and mortality among US adults in two previous, longitudinal studies, possibly indicating lasting effects of psychological distress [6, 24]. Based on our findings, a corollary observation is that these previously reported prospective associations with emergency room department utilization and mortality may be partially mediated by the association between psychological distress and short sleep duration. Thus, interventions that aim to promote mental health and adequate sleep may have beneficial impacts beyond those two factors.

### 4.2. Limitations

The findings are subject to at least four limitations. First, other sleep-related factors (e.g., insomnia, excessive daytime sleepiness, shift work, sleeping pills, and menopause) were not examined. Second, BRFSS data are collected via telephone survey and may not be representative of adults in households without phones. Third, as the data were collected from adults in five states, the generalizability of our findings may be limited. Fourth, the cross-sectional study design does not permit determining temporal sequence of psychological distress with short and long sleep duration and thus cause and effect associations cannot be determined [33].

## 5. Conclusion

In summary, the findings of this study suggest that milder psychological distress, in addition to SPD, was associated with a greater probability of short and long sleep duration. Additionally, some associations for short sleep duration were statistically different for women and men, but not necessarily clinically meaningful. Nevertheless, psychological distress could potentially be targeted through interventions as a way to improve the percentage of women and men who are sleeping sufficiently. Insofar as interventions promote mental health and also encourage adequate sleep, the health impacts of these interventions may extend well beyond the immediate.

## Figures and Tables

**Table 1:** Prevalence of serious psychological distress, short sleep duration, and long sleep duration, by selected sociodemographic characteristics among adults aged ≥18 years in 5 states: Behavioral Risk Factor Surveillance System, 2013.

Characteristic	No.	Serious psychological		Short sleep duration		Long sleep duration	
		distress, %		(≤6 hours), %		(≥9 hours), %	
		(95% CI)	*P* (*χ* ^2^)	(95% CI)	*P* (*χ* ^2^)	(95% CI)	*P* (*χ* ^2^)
Overall	36,859	4.0 (3.7, 4.4)		33.9 (33.1, 34.7)		7.8 (7.3, 8.2)	
Gender							
Men	15,361	3.1 (2.6, 3.6)	<.001	35.7 (34.4, 36.9)	<.001	6.9 (6.2, 7.5)	<.001
Women	21,498	4.9 (4.4, 5.4)		32.3 (31.3, 33.4)		8.6 (8.0, 9.2)	
Age (years)							
18–24	1,707	3.9 (2.7, 5.1)	<.001	31.0 (28.1, 33.9)	<.001	10.8 (8.9, 12.6)	<.001
25–34	3,416	4.9 (3.8, 6.0)		40.5 (38.2, 42.7)		6.2 (5.0, 7.4)	
35–44	4,730	4.4 (3.6, 5.2)		35.5 (33.5, 37.6)		6.0 (4.9, 7.1)	
45–64	15,332	4.7 (4.2, 5.3)		35.4 (34.2, 36.6)		5.8 (5.2, 6.4)	
≥65	11,674	1.5 (1.2, 1.9)		25.5 (24.2, 26.8)		13.0 (12.0, 14.0)	
Race/ethnicity							
White, non-Hispanic	31,506	3.9 (3.5, 4.2)	.09	32.3 (31.4, 33.1)	<.001	7.8 (7.3, 8.2)	.13
Black, non-Hispanic	1,485	5.1 (3.3, 6.9)		43.7 (39.8, 47.5)		8.5 (6.3, 10.6)	
American Indian or Alaskan Native	321	6.6 (2.9, 10.3)		48.9 (40.0, 57.7)		8.7 (4.7, 12.7)	
Asian	629	2.1 (0.5, 3.7)		36.8 (31.5, 42.1)		4.6 (1.9, 7.3)	
Native Hawaiian or Pacific Islander	76	4.3 (0.0, 9.5)		33.9 (20.4, 47.3)		2.8 (0.0, 6.7)	
Other race, non-Hispanic	303	4.6 (1.1, 8.0)		42.2 (32.4, 52.0)		6.9 (2.2, 11.7)	
Multiracial, non-Hispanic	584	6.0 (2.3, 9.6)		40.5 (32.7, 48.2)		12.7 (4.6, 20.8)	
Hispanic	1,955	4.7 (3.3, 6.1)		36.3 (33.3, 39.3)		7.9 (6.1, 9.7)	
Marital status							
Married	20,035	2.6 (2.2, 3.0)	<.001	31.1 (30.1, 32.1)	<.001	7.2 (6.6, 7.8)	.02
Previously married	10,547	6.4 (5.6, 7.1)		39.4 (37.8, 41.0)		8.1 (7.4, 8.9)	
Never married	6,277	5.3 (4.4, 6.1)		35.8 (33.9, 37.7)		8.7 (7.6, 9.7)	
Educational attainment							
Did not graduate high school	2,026	9.8 (7.9, 11.7)	<.001	39.9 (36.8, 43.0)	<.001	10.6 (8.6, 12.6)	<.001
Graduated high school	9,174	4.7 (4.0, 5.4)		36.3 (34.7, 37.9)		8.5 (7.6, 9.5)	
Some college or technical school	11,090	3.7 (3.2, 4.3)		35.1 (33.7, 36.4)		7.1 (6.4, 7.8)	
Graduated college or technical school	14,569	1.2 (0.9, 1.4)		27.7 (26.6, 28.8)		6.6 (6.0, 7.2)	
Annual household income							
<$25,000	7,991	9.9 (8.8, 11.0)	<.001	41.2 (39.4, 43.0)	<.001	9.0 (7.9, 10.1)	<.001
$25,000–$49,999	8,346	2.7 (2.1, 3.2)		35.5 (33.8, 37.2)		8.5 (7.4, 9.6)	
≥$50,000	15,798	1.0 (0.8, 1.3)		29.8 (28.7, 30.9)		6.0 (5.4, 6.6)	
Missing	4,724	5.3 (4.2, 6.4)		32.0 (29.8, 34.3)		9.6 (8.3, 10.9)	
Employment							
Employed	19,135	1.7 (1.4, 2.0)	<.001	35.0 (34.0, 36.1)	<.001	5.2 (4.7, 5.8)	<.001
Unemployed	1,900	10.0 (7.8, 12.1)		39.5 (36.1, 43.0)		10.2 (8.2, 12.2)	
Homemaker/student	2,993	3.9 (2.8, 5.0)		28.2 (25.7, 30.8)		8.4 (7.0, 9.8)	
Retired	10,551	1.6 (1.2, 2.0)		24.6 (23.2, 25.9)		13.0 (11.9, 14.1)	
Unable to work	2,280	25.4 (22.4, 28.5)		53.5 (50.1, 57.0)		12.8 (10.5, 15.1)	
Health insurance coverage	33,112	3.4 (3.2, 3.9)	<.001	32.7 (31.9, 33.5)	<.001		.04

*Note.* Weighted percentages; unweighted numbers. CI = confidence interval.

**Table 2:** Crude and adjusted associations between three forms of psychological distress and short sleep duration among adults aged ≥18 years in 5 states: Behavioral Risk Factor Surveillance System, 2013.

Independent variables	Short sleep duration (≤6 hours)					
	All		Women		Men	
	Crude	Adjusted	Crude	Adjusted	Crude	Adjusted
	PR (95% CI)	PR (95% CI)	PR (95% CI)	PR (95% CI)	PR (95% CI)	PR (95% CI)
Model 1: Serious psychological distress	1.95 (1.82, 2.10)	1.58 (1.45, 1.72)	2.04 (1.86, 2.24)	1.56 (1.40, 1.74)	1.89 (1.68, 2.11)	1.58 (1.39, 1.79)
Model 2: K6 category						
0	1.00	1.00	1.00	1.00	1.00	1.00
1-2	1.15 (1.07, 1.23)	1.16 (1.08, 1.24)	1.29 (1.17, 1.42)	1.31 (1.18, 1.44)	1.06 (0.96, 1.17)	1.06 (0.96, 1.17)
3–5	1.41 (1.31, 1.51)	1.38 (1.28, 1.48)	1.55 (1.40, 1.71)	1.53 (1.38, 1.68)	1.33 (1.20, 1.47)	1.28 (1.16, 1.42)
6–10	1.78 (1.66, 1.92)	1.67 (1.54, 1.80)	2.04 (1.84, 2.25)	1.86 (1.68, 2.07)	1.63 (1.46, 1.82)	1.53 (1.37, 1.70)
11+	2.40 (2.22, 2.59)	2.11 (1.94, 2.30)	2.69 (2.42, 2.99)	2.25 (2.00, 2.54)	2.26 (2.02, 2.53)	2.02 (1.79, 2.28)
Model 3: Scale measure of psychological distress	1.09 (1.08, 1.11)	1.08 (1.07, 1.10)	1.11 (1.09, 1.13)	1.10 (1.08, 1.11)	1.08 (1.06, 1.10)	1.07 (1.05, 1.09)
Scale measure of psychological distress^2^	0.998 (0.997, 0.999)	0.998 (0.997, 0.999)	0.997 (0.997, 0.998)	0.997 (0.997, 0.998)	0.998 (0.998, 0.999)	0.999 (0.998, 1.000)

Note. CI = confidence interval; PR = prevalence ratio.

Model 1 examined psychological distress dichotomized (serious/no serious psychological distress), Model 2 examined psychological distress categorized, and Model 3 examined the scale measure of psychological distress (0–24) and included a quadratic term for the scale measure of psychological distress. Age, gender, race/ethnicity, marital status, educational attainment, annual household income, employment, and health insurance coverage are included in all adjusted models. Gender is excluded from the stratified models.

**Table 3:** Crude and adjusted associations between three forms of psychological distress and long sleep duration among adults aged ≥18 years in 5 states: Behavioral Risk Factor Surveillance System, 2013.

Independent variables	Long sleep duration (≥9 hours)					
	All		Women		Men	
	Crude	Adjusted	Crude	Adjusted	Crude	Adjusted
	PR (95% CI)	PR (95% CI)	PR (95% CI)	PR (95% CI)	PR (95% CI)	PR (95% CI)
Model 1: Serious psychological distress	1.73 (1.34, 2.22)	1.39 (1.08, 1.79)	1.64 (1.18, 2.27)	1.37 (1.00, 1.86)	1.78 (1.24, 2.55)	1.43 (0.96, 2.16)
Model 2: K6 category						
0	1.00	1.00	1.00	1.00	1.00	1.00
1-2	0.93 (0.80, 1.07)	0.93 (0.80, 1.07)	0.90 (0.75, 1.08)	0.88 (0.74, 1.06)	0.93 (0.74, 1.18)	0.99 (0.78, 1.25)
3–5	1.02 (0.85, 1.21)	1.00 (0.837, 1.193)	1.11 (0.91, 1.36)	1.07 (0.87, 1.32)	0.87 (0.64, 1.18)	0.92 (0.68, 1.24)
6–10	1.14 (0.94, 1.38)	1.04 (0.852, 1.278)	1.05 (0.84, 1.31)	0.96 (0.76, 1.20)	1.23 (0.88, 1.72)	1.21 (0.86, 1.72)
11+	1.52 (1.22, 1.90)	1.22 (0.969, 1.543)	1.52 (1.14, 2.02)	1.25 (0.94, 1.65)	1.43 (1.03, 1.99)	1.21 (0.83, 1.78)
Model 3: Scale measure of psychological distress	1.03 (1.02, 1.04)	1.02 (1.00, 1.03)	1.03 (1.01, 1.05)	1.02 (1.00, 1.03)	1.03 (1.01, 1.05)	1.02 (0.99, 1.04)

Note. CI = confidence interval; PR = prevalence ratio.

Model 1 examined psychological distress dichotomized (serious/no serious psychological distress), Model 2 examined psychological distress categorized, and Model 3 examined the scale measure of psychological distress (0–24). Age, gender, race/ethnicity, marital status, educational attainment, annual household income, employment, and health insurance coverage are included in all adjusted models. Gender is excluded from the stratified models.
